# Supplementary material for: Double-layer optimization model for integrated energy system under multiple robustness
Source: PLoS One. 2023 Sep 26;18(9):e0291787. doi: 10.1371/journal.pone.0291787 (PMC10522003; doi:10.1371/journal.pone.0291787)
Supplement: Attachment — Submitted filename: Authors Response to Reviewers Comments.docx [file pone.0291787.s002.docx]

**RE: PONE-D-23-15193**

Dear Editor: Sani Isah Abba

We would like to thank the editor for your letter and the comments from the referees about our paper submitted to ***PLOS ONE* (PONE-D-23-15193)** and giving us a chance to revise the paper. We also thank the reviewers for giving us constructive suggestions which would help us in depth to improve the quality of the paper. Here we submit a new version of our manuscript with the title “Double-layer Optimization Model for Integrated Energy System under Multiple Robustness”, which has been modified according to the editor’s suggestions. We mark all the changes in red in the revised manuscript.

Sincerely yours,

Xiaobao Yu.

-------------------------------------------------------------------------------------------------------

The following is a point-to-point response to reviewers’ comments.

**Response to reviewers:**

**Reviewer #1:**

1. The abstract should contain research gaps, novelties, contributions and main results.

Authors’ Answer: Thanks to the valuable opinions of the experts, the author has rewritten the abstract as follows:

Output instability is one of the important constraints limiting the large-scale application of renewable energy. The development of comprehensive energy systems can effectively improve energy utilization efficiency, but there is still a problem of randomness in renewable energy output. The paper conducts research on the uncertainty of distributed energy output and load, constructs a comprehensive energy system optimization model that takes into account the robustness of bi-level programming, and solves the model using the firefly algorithm. The calculation results show that optimizing uncertainty can significantly reduce the actual operating costs of the system, with a maximum reduction of 14.43%. When the distributed wind power interval is within [0190], a dynamic balance between cost and consumption rate can be achieved.

2. For such an international journal, you should provide the research globally not for China. The information about the China should be moved to be as case study.

Authors’ Answer: Thanks to the valuable opinions of the experts, the author has included the specific situation of China in the case study, please refer to the red section of the paper for details.

3. The research fall into the concepts of "energy management", "Energy efficiency", and "energy awareness". In case these are neglected by the authors, the paper is not on the frontier of the concept and should be purged from publication. a quick scan of the google.scholar with the following keywords, reveals a plenty of research in this regard: "energy awareness"+"robust optimization" and "energy efficiency"+"robust optimization"+"smart". Without considering these papers, the paper should be purged from publication because it is not on the frontier of the concept.

Authors’ Answer: Thanks to the valuable opinions of the experts, the author conducted a literature search based on the combination of "energy awareness" + "robust optimization" and "energy efficiency" + "robust optimization" + "smart", and updated the literature review section. Please refer to the highlighted section of the paper for details.

4. What are the contributions, research questions, and gaps?

Authors’ Answer: Thanks to the valuable opinions of the experts, the contributions of this paper are as follow: based on the bilateral uncertainty of integrated energy system, an optimization model of integrated energy system is constructed, considering the robustness of bi-level programming; considering the output uncertainty, a multi-objective optimization model of integrated energy subsystem is constructed based on Nash equilibrium; considering the uncertainty of load and energy price, an integrated energy subsystem optimization model is constructed based on stochastic chance constrained programming.

The research questions and gaps are to consider the constraints of uncertainty on the large-scale application of renewable energy, which results in poor performance in comprehensive energy systems. In the context of the contradiction between carbon emissions reduction and system operating costs, how to minimize the uncertainty of supply and demand, and find a dynamic balance between the proportion of renewable energy sources and operating costs is one of the urgent issues to be studied.

5. You use decentralized system. Why? Using some research papers, you should justify the use of such a system through bi-level programming.

Authors’ Answer: Thanks to the valuable opinions of the experts, distributed energy has strong flexibility and is very advantageous for the regulation of integrated energy systems. The paper adopts the double-layer optimization theory to optimize the operation of integrated energy systems considering multiple uncertainties. Currently, this method is a relatively mature theory and can effectively solve multi-objective optimization problems, For example, in the upper level model of the paper, the multi-objective function is to minimize the operating cost of the electrical energy subsystem and maximize the consumption rate of renewable energy. The uncertainty of wind power, photovoltaic power, and load in the electrical energy subsystem is described in interval form, and a game is set up between the system decision-maker and the renewable energy output decision-maker. By solving the Nash equilibrium, the optimal collaborative solution of the upper level model is obtained. The lower level model adopts stochastic chance constrained programming theory for modeling, considering the uncertainty of energy prices, and describing the objective function and constraint conditions in probability form.
